# SimKinet: A free educational tool based on an electrical analogy to solve chemical kinetic equations

**DOI:** 10.1371/journal.pone.0213302

**Published:** 2019-03-08

**Authors:** Manuel Caravaca, Pilar Sanchez-Andrada, Antonio Soto-Meca

**Affiliations:** 1 Department of Sciences and Computing, University Centre of Defence, Spanish Air Force Academy, Santiago de la Ribera, Murcia, Spain; 2 Department of Organic Chemistry, Faculty of Chemistry, University of Murcia, Regional Campus of International Excellence "Campus Mare Nostrum", Espinardo, Murcia, Spain; Instituto Nacional de Astrofisica Optica y Electronica, MEXICO

## Abstract

In this article we introduce the software SimKinet, a free tool specifically designed to solve systems of differential equations without any programming skill. The underlying method is the so-called Network Simulation Method, which designs and solves an electrical network equivalent to the mathematical problem. SimKinet is versatile, fast, presenting a real user-friendly interface, and can be employed for both educational and researching purposes. It is particularly useful in the first courses of different scientific degrees, mainly Chemistry and Physics, especially when facing non-analytic or complex-dynamics problems. Moreover, SimKinet would help students to understand fundamental concepts, being an opportunity to improve instruction in Chemistry, Mathematics, Physics and other Sciences courses, with no need of advanced knowledge in differential equations. The potency of SimKinet is demonstrated via two applications in chemical kinetics: the photochemical destruction of stratospheric ozone and the chaotic dynamics of the peroxidase-oxidase reaction.

## Introduction

Differential equations allow a very convenient modelling of some essential natural phenomena that evolve over time in a continuous way. This is a prime reason why differential equations are ubiquitous in so many scientific and technological disciplines [1–3]. In this context, knowing how to pose and solve a particular set of differential equations is a crucial issue in order to describe a wide range of fundamental processes, as well as to recognize and control the variables governing them.

Not only researchers and qualified professional, but also students, who will frequently be faced with the learning of phenomena of diverse nature in various scientific disciplines, require of this knowledge. A multitude of examples can be found in very diverse scientific areas at undergraduate levels, for instance, the damped and forced oscillator, the coupled pendulum, diffusion phenomena, RLC electrical circuits, radioactive decays, bacterial growth, predator-prays models [3], or time evolution of atmospheric pollutants [4].

One matter requiring frequently of the analysis and solution of differential equations is Physical Chemistry. This branch of Chemistry is concerned with how, why and when of the chemical reaction. Chemical kinetics and reaction dynamics, fundamental subfields of Physical Chemistry, deal with the rates of processes and with how reactions take place. Chemical reactions govern our environment and pollutants evolution, life processes, food production, energy power and many other industrial processes designed to produce all kinds of goods indispensable for our life as we know it nowadays. We are in daily contact with fragrances, food additives, medicines, detergents and surfactants, dyes and pigments, plastics, elastomers, synthetic fibers, agrochemicals, etc., just to put a few examples, along with a myriad of products and new materials with important technological applications [5–7].

Chemical kinetics and reaction dynamics are not only a central intellectual cornerstone of Chemistry [8,9], but they become essential to gain a deep understanding of the chemical reaction and to get control over the products composition and the rates which they are obtained. To this end, it is mandatory to elucidate and understand the kinetics of the chemical processes by means of coupled differential equations [10–15].

In this context, the mathematical resolution of kinetic differential equations is often not a trivial task, and in many cases it becomes necessary the use of approximations, both theoretical, such as the well-known steady stationary approximation (SSA) [16–18], and numerical, generally implemented via computational algorithms such as the Runge-Kutta method (RKM) [3]. Recently, the authors have described how to solve a complex system of differential kinetic equations via numerical simulations [19] by means of the so-called Network Simulation Method (NSM), a numerical approach which employs an electrical analogy, being topologically equivalent to the original mathematical problem. This method is well-spread and efficient, showing applications in many scientific areas, and specifically in Chemistry [20]. The results were very satisfactory, but they required advanced numerical and programming skills, as well as deep knowledge in electrical circuits. Then, the authors realized that making available this approach for students and researchers would demand the design of a software with specific requirements, including: (a) a user-friendly interface which allows a simple way to introduce the system of differential equations; (b) a black box structure, with no need of programming or advanced numerical skills; (c) versatility, in order to solve kinetic problems and also other kind of differential equations associated with scientific phenomena; (d) a didactic focus, which allows the use of computer simulations to learn central concepts in Chemistry or Physics courses by means of guided-inquiry activities; (e) free software.

With these goals in mind we have developed SimKinet, a software which can be employed for students to solve a wide range of problems involving first-order ordinary differential equations. Besides, SimKinet can also help students to understand fundamental concepts, taking advantage of computing-learning applied to improve instruction in Chemistry, Mathematics, Physics and other Science courses [21–23], even regarding the possibility of carrying out low-cost laboratory practices [24]. A learning environment with computer simulations allows the student to confirm predictions or to test hypotheses, thus deeping in the conceptual background of the scientific phenomena. Nowadays, SimKinet is employed for the subjects Physics and Chemistry of first course of Industrial Engineering degree in the University Centre of Defence at the Spanish Air Force Academy to broaden the study of Kirchhoff’s laws and chemical kinetics, respectively. It has also been employed for Final Degree Projects, to determine the kinetic-thermodynamic switching point in a tandem of pericyclic reactions and to follow the dynamics of pursuit-predator aircraft models [25,26].

In this work we introduce SimKinet along with some interesting applications in chemical kinetics, showing its strength for solving advanced numerical problems. Although a wide spectrum of software designed to solve kinetic chemical equations is available, the most recognized among them are not free [27,28], while others present restrictions in the timestepping [29,30], considered as fixed, or demand additional computing skills [31]. Due to its simple handling and adaptative timestepping, SimKinet becomes an outstanding alternative.

With the aim of clarifying the numerical approach underlying this software, in section “The electrical analogy” we introduce the basis of the electrical analogy and the NSM. In section “The SimKinet software”, the user-friendly SimKinet interface is shown via a prototypical chemical reaction. In addition, in section “Applications” we show the educational and researching capabilities of SimKinet through two particular examples: a kinetic system of differential equations with no analytical solution, the Chapman model, and a chemical system with chaotic dynamics, known as the Olsen attractor. Additionally, a test with numerical methods implemented in software Mathematica [32] and MATLAB [33] has been performed in order to check the accuracy of SimKinet.

## The electrical analogy

The electrical analogy of scientific problems is well recognized as a very useful and attractive educational subject, which constitutes a standard procedure in some undergraduate textbooks [34]. This approach is no more than a formal equivalence between the governing equations of the problem and an electrical network. In this analogy, the terms of the original equations are usually identified with appropriate electrical devices. Within this framework, the mechanical-electrical analogies are well-known since James Clerk Maxwell’s era [35]. A classical example, found in the subjects Physics and Mechanics belonging to first courses in Physics, Chemistry and Engineering degrees, is the equivalence between a non-forced damped oscillator and an electric circuit [36]. The differential equation that describes the original problem, through Newton’s second law of motion, is:

$$m\frac{{\text{d}}^{2}x}{\text{d}t^{2}}+b\frac{\text{d}x}{\text{d}t}+kx=0$$ (1)

where *m* is the mass, *x* is the position coordinate along the X axis, *b* is the damping constant and *k* is the restoring constant. On other hand, Kirchhoff’s second law for electric circuits can be easily applied to a series RLC circuit [37], thus obtaining:

$$L\frac{{\text{d}}^{2}q}{\text{d}t^{2}}+R\frac{\text{d}q}{\text{d}t}+\frac{1}{C}q=0$$ (2)

where *L* is the inductance, *q* is the electrical charge, *R* is the resistance and *C* is the capacitance of the electric circuit, respectively. Eqs (1) and (2) present the same mathematical structure so, by establishing the particular equivalence *m* → *L*, *b* → *R*, *k* → 1/*C* and *x* → *q*, the analogy is completed. Then, the dynamics of the system can be followed through the resolution of Newton’s or Kirchhoff’s laws, equivalently. This particular example is easy to solve theoretically in both cases, and usually presents strictly academic interest. However, in some other problems, the use of the electrical analogy allows an easier handling of the theoretical solution, which can be particularly valuable in Lagrangian dynamics, when dealing with many-body systems [34,38]. Furthermore, the electrical analogy can be extended to other scientific areas, such as heat transfer, fluid flow, diffusion or chemical reactions [20,39,40].

The aim of the analogy is always building an electrical network equivalent to the original equations, which can be carried out in different ways, thus leading to different electrical analogies [20,41]. The NSM becomes an outstanding alternative, because it makes use of an electrical analogy which employs very few and simple electrical devices to build the equivalent circuit [20].

As far as the chemical kinetics is concerned, the full kinetic study of a chemical process frequently comprises a set of coupled first order differential equations. Each species has an associated equation, where the time variation of its concentration, [*x*_*i*_], is related with those of the other species and a set of kinetic constants, *k*_*j*_, *j* = 1, *m*, as shown in Eq (3):

$$\frac{\text{d}[x_{i}]}{\text{d}t}=f(\left[x_{1}],[x_{2}]\ldots [x_{n}\right])$$ (3)

Frequently, the whole mathematical problem does not present analytical solution [19]. This fact requires either theoretical approximations or numerical methods. Among the latter, numerical methods such as the 4th order RKM generally give accurate results, except in several circumstances when dealing with complex kinetic systems. For example, kinetic schemes involving multiple reaction steps whose values of rate constants show quite different orders of magnitude [9,11,42–45]. A simple case can be found when the rate constant value for the step exerting the strongest effect on the overall reaction rate is very low.

To illustrate how the running time of a simulation can be affected by the value of the rate constant corresponding to this step, *k*_R_, consider the case in which it has frequency units (1/s). Consequently, the corresponding reaction step must take place once every 1*/k*_R_ seconds. When *k*_R_ is too small, standard simulation algorithms consume very large CPU times to reach the steady state of the system [19]. In particular, in such circumstances the RKM becomes too slow, being desirable to employ alternative numerical algorithms, which, in general, require advanced programming skills [46].

In those situations, where traditional algorithms may be inefficient, the electrical analogy introduced by the NSM and implemented in SimKinet becomes particularly useful, because of its simple circuit design that can take advantage of the powerful capabilities of modern circuit simulation computer codes, which employ the more complex algorithms of calculus. Since the electrical analogy creates an equivalent electrical circuit from the original differential equations, to complete the NSM the numerical problem has to be solved via an appropriate circuit software. The choice for SimKinet has been PSpice [47], due to its well-know numerical efficiency [39] and the avaliability of a free version, which has to be installed previously.

In the next section we shall introduce the basis of the NSM, directly appling the analogy to chemical reactions.

### The Network Simulation Method. Application to chemical reactions

The numerical procedure known as the Network Simulation Method, which is properly based on the electric analogy of the transport process, is capable of solving the set of differential equations associated with a chemical process in a time orders of magnitude lower than the traditional algorithms, presenting an advantage in terms of scientific computing [48,49]. The method comprises the design and solution of an electric circuit formally equivalent to the original set of differential equations. However, this is not a trivial task. The general method involves establishing the following equivalences: *I* (electric current, in W/m^2^) → flow variable of the problem; *V* (electric potential, in V) → potential variable of the problem.

From the point of view of the network model, each equation of the set of differential equations is considered as a circuit described by Kirchhoff’s current law (KCL). The complete set of equations is equivalent to a global electric network containing as many electric circuits as equations [20]. For chemical kinetic equations as Eq (3), the electrical devices employed by the NSM to model each differential equation are: (a) a capacitor, associated with the first derivative of the concentration in Eq (3); (b) voltage-controlled current sources, which easily implements the coupling of the equations, and whose value can be defined as the function *f*, whatever its expression, in the right side of Eq (3); (c) a resistor of high value, only employed to guarantee criteria of continuity.

The NSM electrical analogy between a set of kinetic coupled differential equations and an electric circuit network can be established as follows. Let us start by applying the equivalence to the particular case of a one-step chemical reaction, whose balanced equation can be expressed in the following way:

$$\sum_{i=1}^{n}a_{i}R_{i}\overset{k_{\text{F}}}{\underset{k_{\text{R}}}{↔}}\sum_{j=1}^{m}b_{j}P_{j},$$ (4)

where *a*_*i*_ and *b*_*j*_ are the stoichiometric coefficients belonging to reactant *R*_*i*_ and product *P*_*j*_, respectively. The rate constant for the forward reaction is denoted by *k*_F_, whereas *k*_R_ is that for the reverse step.

Since the number of moles of these species, $n_{R_{i}}$ and $n_{P_{j}}$, are proportional to their stoichiometric coeffcients, we can define a quantity *dξ* which is, in general, a function of time, but equal in magnitude for all reactants and products:

$$d\xi ={d\xi }_{R_{i}}={d\xi }_{P_{j}},\;\forall \;i,j$$ (5)

$${d\xi }_{R_{i}}=-\frac{dn_{R_{i}}}{a_{i}},\;{d\xi }_{P_{j}}=\frac{dn_{P_{j}}}{b_{j}}\;.$$ (6)

The reaction rate *R* at constant volume can be defined for i-th species as:

$$R=-\frac{1}{a_{i}v}\frac{dn_{R_{i}}}{dt}=\frac{1}{b_{j}v}\frac{dn_{P_{j}}}{dt}=\frac{1}{v}\frac{d\xi }{dt},$$ (7)

Where *v* is the volume of the reaction. We can state from Eqs (5) and (6) that *R* is independent of the chosen species, but it is a time-varying function.

Now we define $J_{R_{i}}$ and ${J^{*}}_{R_{i}}$ as the standard and renormalized flow of the reactant *R*_*i*_ as follows (equivalent for products, not shown):

$${\;J}_{R_{i}}=-\frac{dn_{R_{i}}}{dt}$$ (8)

$${J^{*}}_{R_{i}}=\frac{J_{R_{i}}}{a_{i}}=\frac{d\xi_{R_{i}}}{dt}.$$ (9)

It is more suitable to write Eq (9) and its equivalent for products in terms of the concentration of the species, which defines the electric currents that establish the analogy between the chemical and electrical systems:

$${{\;J}^{*}}_{R_{i}}=-\frac{dn_{R_{i}}}{a_{i}dt}=-\frac{v}{a_{i}}\frac{d[R_{i}]}{dt}$$ (10)

$${J^{*}}_{P_{j}}=\frac{dn_{P_{j}}}{b_{j}dt}=\frac{v}{b_{j}}\frac{d[P_{j}]}{dt}.$$ (11)

In these expressions, [*R*_*i*_] and [*P*_*j*_] are the concentrations of the *i*- and *j*-th reactant and product, respectively. From Eqs (5) and (6) it is depicted that:

$${{J^{*}=J}^{*}}_{R_{i}}={J^{*}}_{P_{j}},\;\forall \;i,j.$$ (12)

This last relation is the mass local balanced equation corresponding to *R*_*i*_ and *P*_*j*_, which is a process of creation-annihilation. From Eq (12):

$${{J^{*}-J}^{*}}_{R_{i}}=0,\;\forall \;i,$$ (13)

which remains valid for products. That is, each species will follow an equation such as Eq (13).

From the point of view of the network model, the last equation can be considered as Kirchhoff’s current law, being equivalent to the corresponding differential equations describing the evolution of the concentration of the chemical species over time [20]. Each chemical species presents a flow term ${J^{*}}_{R_{i}}$, whose theoretical expressions, Eqs (10) and (11), resemble the expression of the current intensity *I*_*c*_ at the ends of a capacitor: *I*_*c*_ = *C*d*V*/d*t* (*C* and *V* being the capacitance and voltage at the ends of the device). Because of this similarity, the first order derivative of the concentration inside each differential equation, Eq (4), is modeled via a capacitor, whose voltage *V*_*i*_ is then equivalent to [*R*_*j*_] (analogous for products). The remaining term of Eq (13), J*, which was unknown at the beginning, is now easy to determine, since each differential equation has to satisfy the KCL, because they constitute mass local balanced equations. Examining each differential equation, J* just represents the remaining addends of the equation, implemented via voltage-controlled current sources. The application of the method to a prototypical reaction is shown in the S1 Appendix.

The generalization of the NSM approach to a multi-step chemical reaction can be performed in an equivalent way.

In this way, SimKinet has been designed to automatically implement the NSM for solving a system of differential equations corresponding to any chemical kinetic scheme. The software creates the equivalent electric network whatever be the terms of the equation and their expressions, and directly run the model in the electric circuit software PSpice. For that, it employs a numerical algorithm which combines the trapezoidal method and a modified Gear method, both of them of 2^nd^ order with variable time-stepping [49]. The user does not need to manage the mathematical equations involved since this work is done by PSpice.

The interface communication of SimKinet is immediate and user-friendly through the window environment created in the visual C# source code. The resulting simulation data can be graphically displayed in the SimKinet environment or using MATLAB, an even can be exported as data files, due to appropriate routines implemented in the software. Besides, SimKinet is able to cover a wide range of applications involving systems described by first order ordinary differential equations.

## The SimKinet software

SimKinet satisfies two basic requirements for simulating kinetic chemical differential equations. Firstly, it has a simple, editable and visual environment allowing an easy management. This enables the user to understand the sequential steps taking place in the simulation. Secondly, the program is fast and reliable, thus offering numerical advantages over traditional simulation algorithms. When employed for academic purposes, once students have solved a series of simple educational problems, they can naturally evolve into more complex systems.

In general, we must define in SimKinet as many equations as participant species. The differential equation associated to the *n*-th species involved in a chemical reaction has the following general form [20]:

$$\frac{d{[x}_{n}]}{dt}=\sum_{i}k_{i}\prod_{j}{\left[x_{ij}\right]}^{\alpha_{ij}}$$ (14)

where [*x*_*n*_] is the concentration of the *n*-th species, [*x*_*i*,*j*_] is the concentration of species involved in the reaction, *k*_i_ is the kinetic rate constant of addend *i* and *α*_*ij*_ is an appropriate stochiometric coefficient. To complete the mathematical model we must also specify the initial concentrations of all species, i. e., [*x*_*n*_](t = 0).

**Fig 1:**
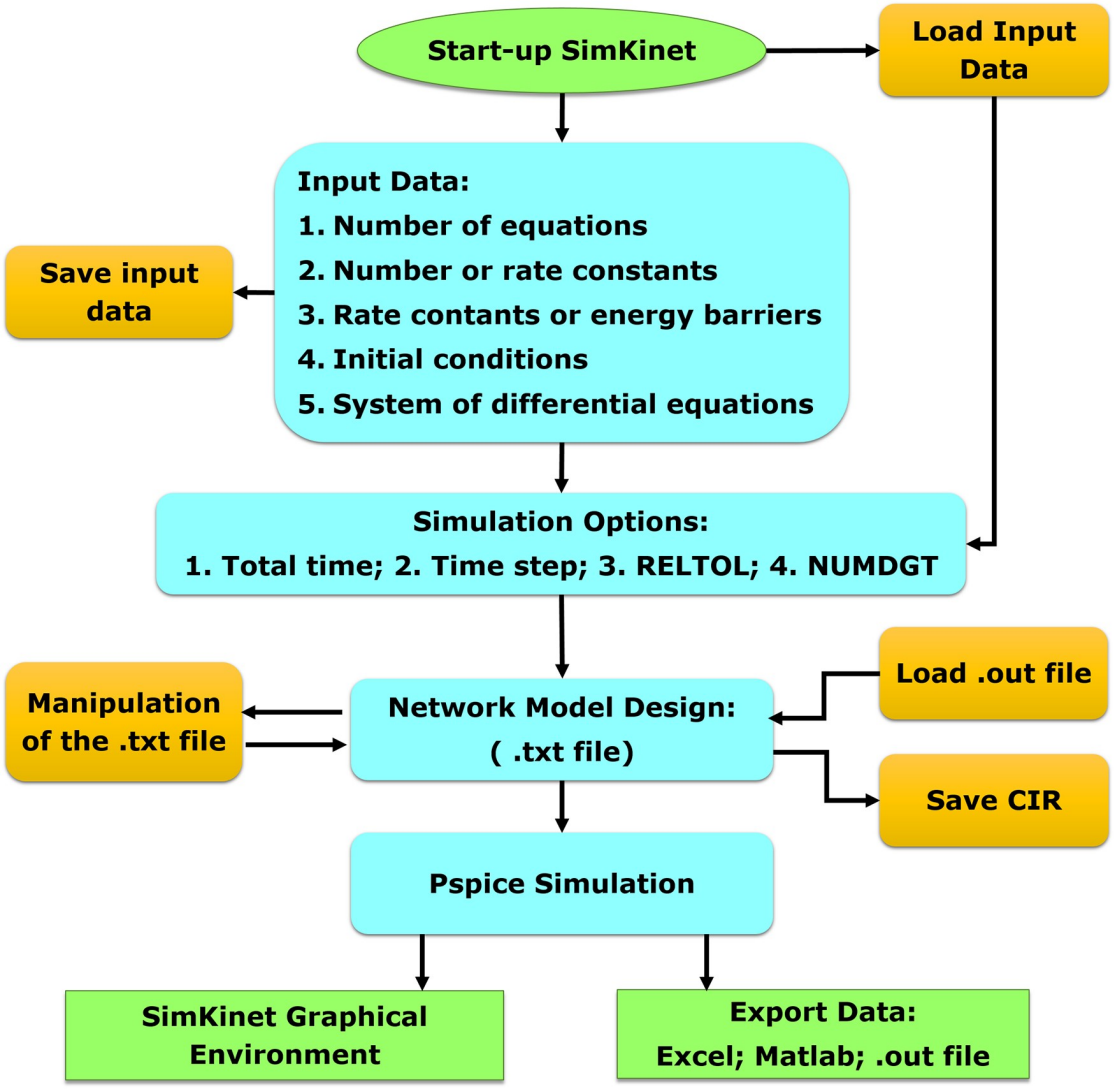
Simplified flow diagram for the software SimKinet.

Once finished the data input process, the program automatically designs the network model, which can be handled by the user before the simulation, if necessary. Then, the system performs the numerical simulation in PSpice and displays a window where the user can perform graphical representations. Besides, the data can be exported in Excel or MATLAB formats for further manipulation by suit the user. To outline the overall working of SimKinet, we show in Fig 1 the simplified flow diagram of the software.

**Fig 2:**
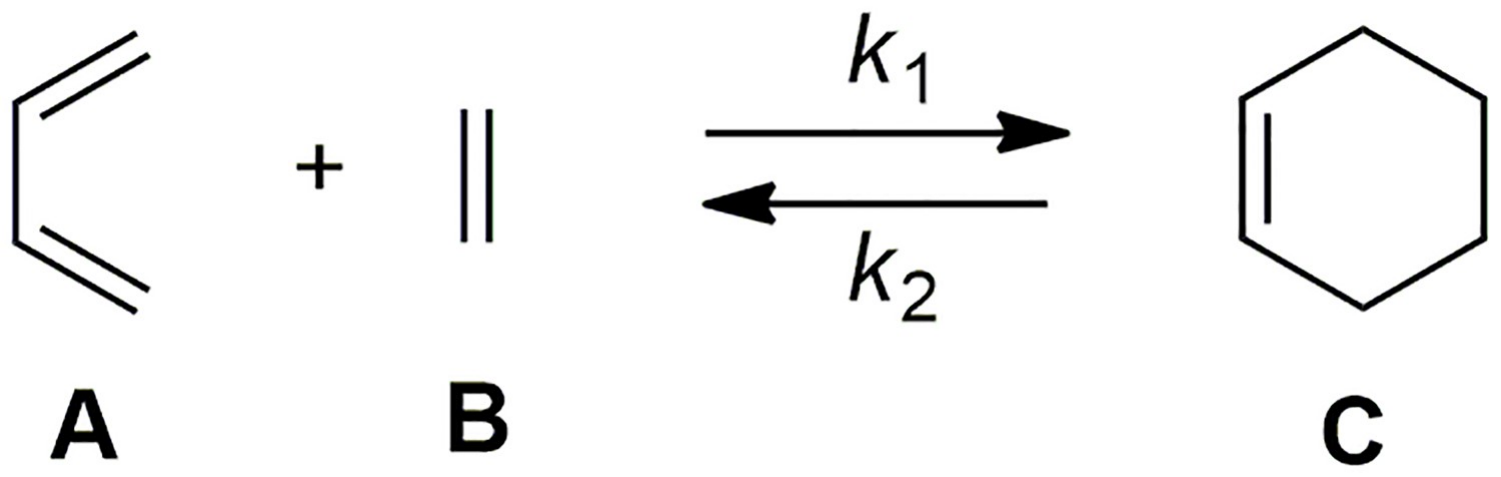
Diels-Alder cycloaddition reaction between 1,3-butadiene and ethene leading to cyclohexene showing the rate constants for the forward and reverse processes.

In the following, we will illustrate the use of SimKinet, step by step, through a prototypical organic reaction: the Diels-Alder cycloaddition reaction between *s*-cis-1,3-butadiene and ethene leading to cyclohexene. This chemical process corresponds to a single kinetic scheme as shown in Fig 2.

Where **A** (*s*-cis-1,3-butadiene), **B** (ethene) and **C** (cyclohexene) are the chemical species involved in the process, and *k*_1_ and *k*_2_ are the corresponding forward and reverse rate constants, respectively. The set of differential equations associated with the chemical reaction are:

$$\begin{array}{l} \frac{\text{d}\left[A\right]}{\text{d}t}=\frac{\text{d}\left[B\right]}{\text{d}t}=-\frac{\text{d}\left[C\right]}{\text{d}t}\\ \frac{\text{d}\left[A\right]}{\text{d}t}=-k_{1}\left[A\right]\left[B\right]+k_{2}\left[C\right] \end{array}$$ (15)

with the following initial conditions: ${\left[A\right]}_{0}={\left[B\right]}_{0}=1\frac{mol}{l},\;{\left[C\right]}_{0}=0\;\frac{\text{mol}}{\text{l}}$.

### Input data and network design

**Fig 3:**
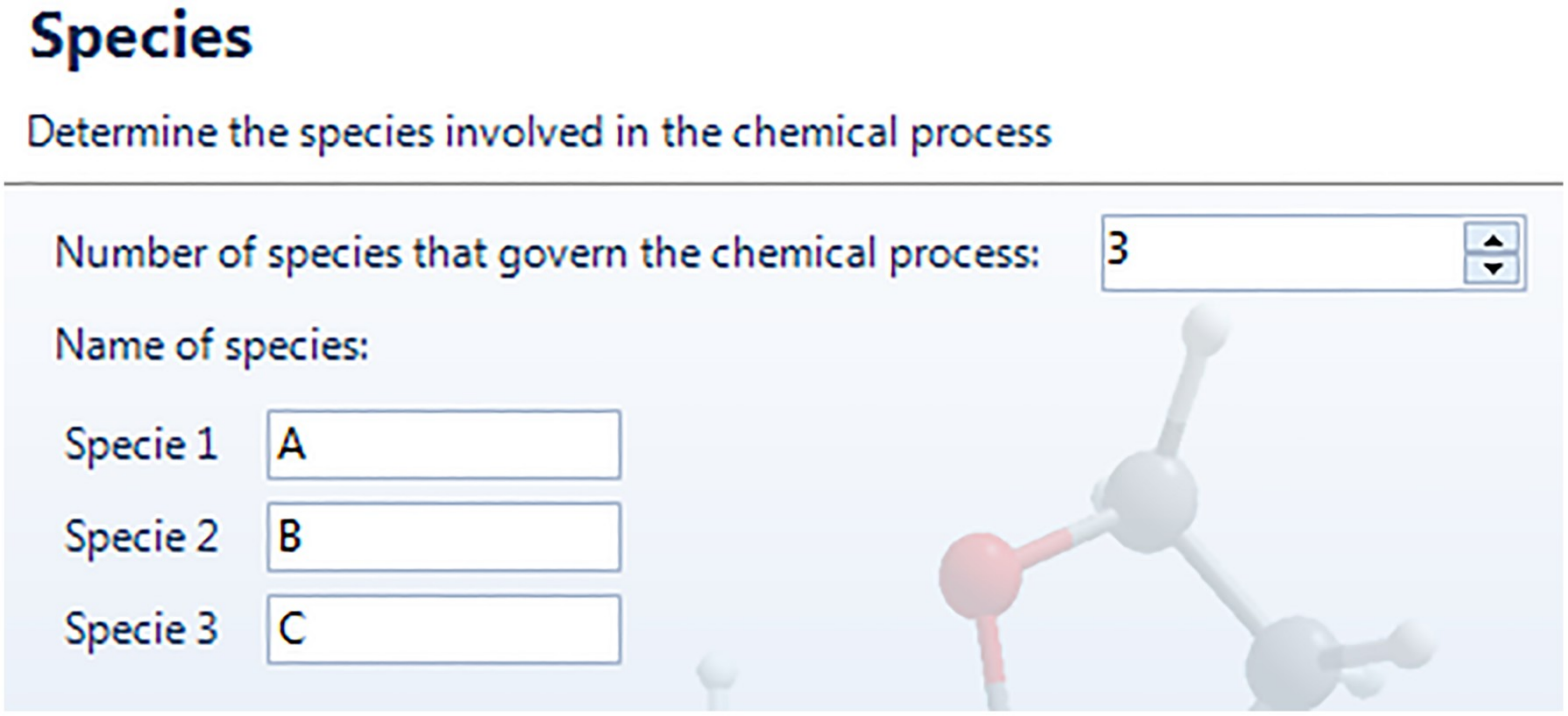
Window-dialog screen to introduce the number and names of the participant chemical species.

Firstly, we should define the number and name of each species involved in the chemical process. The first dialog window of input data is called “Species”, shown in Fig 3 for the academic problem defined in Eq (15).

**Fig 4:**
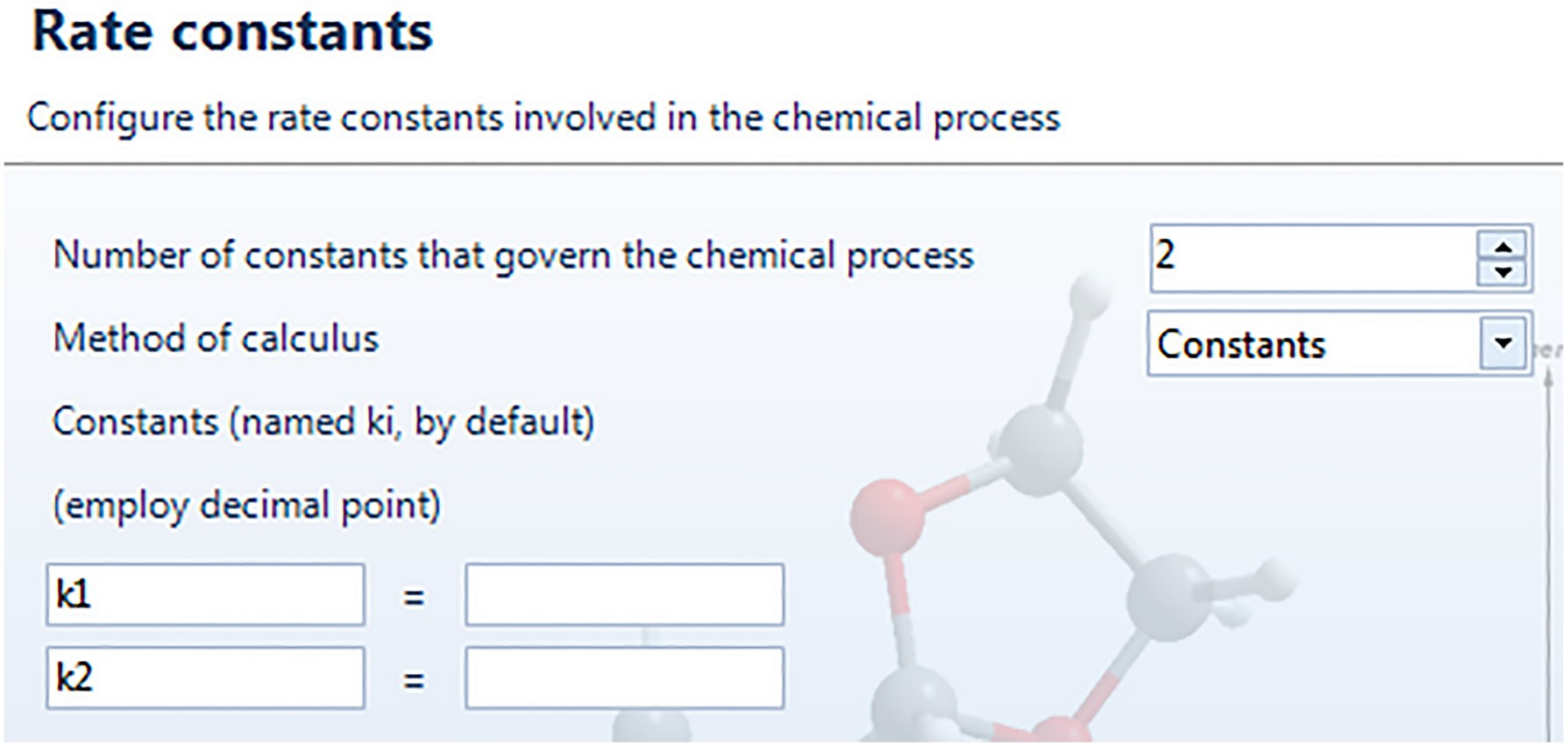
Window-dialog screen to introduce the number, names and values of the rate constants. For this option, the values are directly introduced.

**Fig 5:**
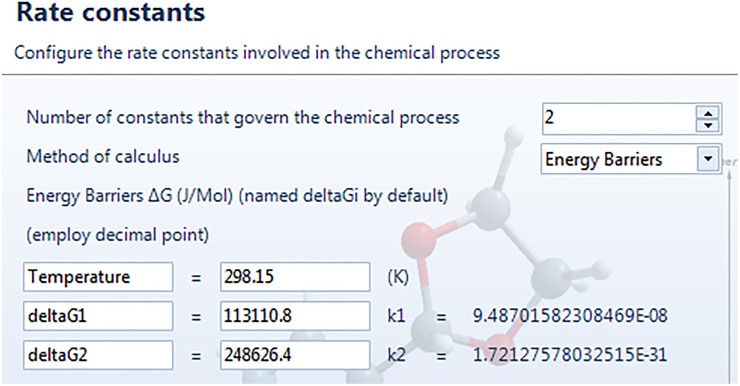
Window-dialog screen to determine the values of the rate constants via the input of the energy barriers corresponding to the kinetic profile of the reaction.

The next window, “Rate constants”, deals with the kinetic rate constants of the chemical reaction. By choosing “constants” from the dropdown menu squared in red in Fig 4, which is the option by default, we can introduce the name and value of each rate constant. If the tab “Energy Barriers” is selected, the values of the free energy barriers associated to each step have to be introduced, as shown in Fig 5. We can customize the name of the barriers and, once the values are entered, the corresponding rate constants are calculated in real time by means of Eyring’s equation [50] and displayed on the screen (orange box). We will follow this latter procedure for the Diels-Alder reaction, by introducing the energy barriers corresponding to the kinetic profile, equal to Δ*G*_1_ = 113110.8 J·mol^-1^ and Δ*G*_−1_ = 248626.4 J·mol^-1^ (see Fig 5) [51].

**Fig 6:**
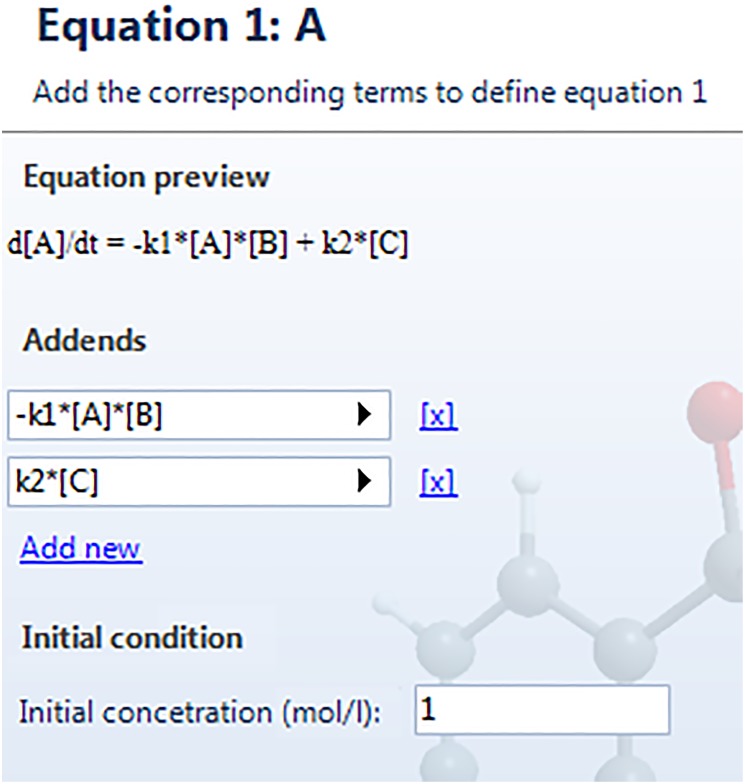
Definition of the differential equation associated to a generic species.

**Fig 7:**
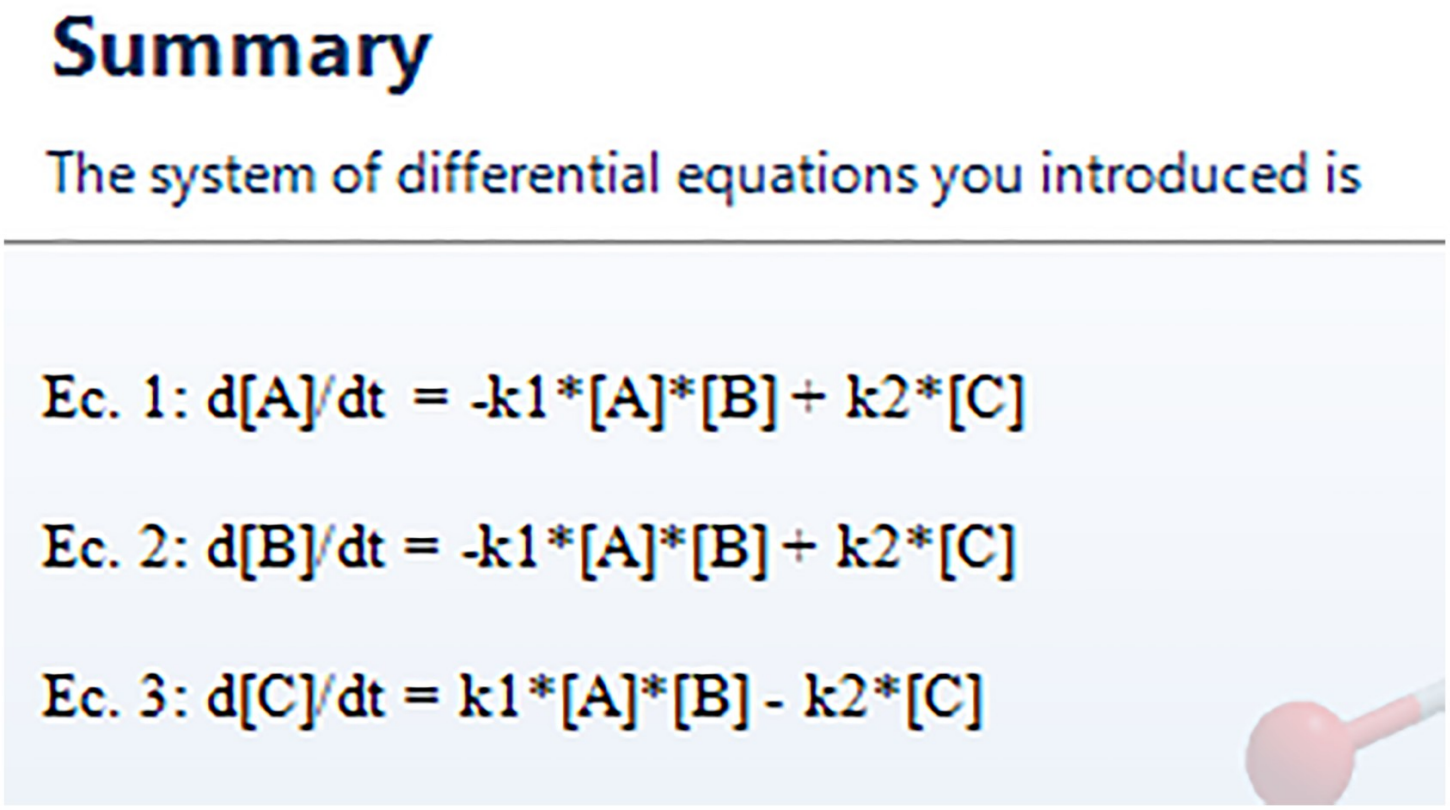
Summary window, which shows the complete set of differential equations corresponding to the chemical profile.

The system of differential equations has to be defined in subsequent windows. It will appear as many windows as species we initially selected. The corresponding equation for species A is called “Eq 1: A” and has the form depicted in Fig 6. In “Equation preview” we can see at any time the appearance of the corresponding differential equation. To include new addends to the equation “Add new” has to be clicked. For our example, the differential equation is configured with two addends, and the complete result is shown in the preview (see Fig 6). The dialog box “initial condition” sets the initial concentration of the compound, in mol/l, which corresponds to the initial condition of the differential equation. The equations associated to the remaining species can be defined by pressing the “Next” button. Similar windows to Fig 6 will appear on the screen. Once the system of differential equations is defined, we can check each equation in the next window, “Summary”, but at this point we cannot change any setting. In case of error, we can go back by clicking the button “Previous”. The summary of Eq (15) is shown in Fig 7. It is possible to save all the steps taken so far by clicking the button “Save system” in the lower left corner on this last window. The extension of the saved file is .eq, and can be loaded in subsequent executions from the “Species” window (corresponding to Fig 3) by pressing the button “Load from file”, located on the lower left region. All parameters and equations will be loaded, and the user will just move through the windows by pressing the “Next” button until reaching “Summary”, changing any input data if necessary.

**Fig 8:**
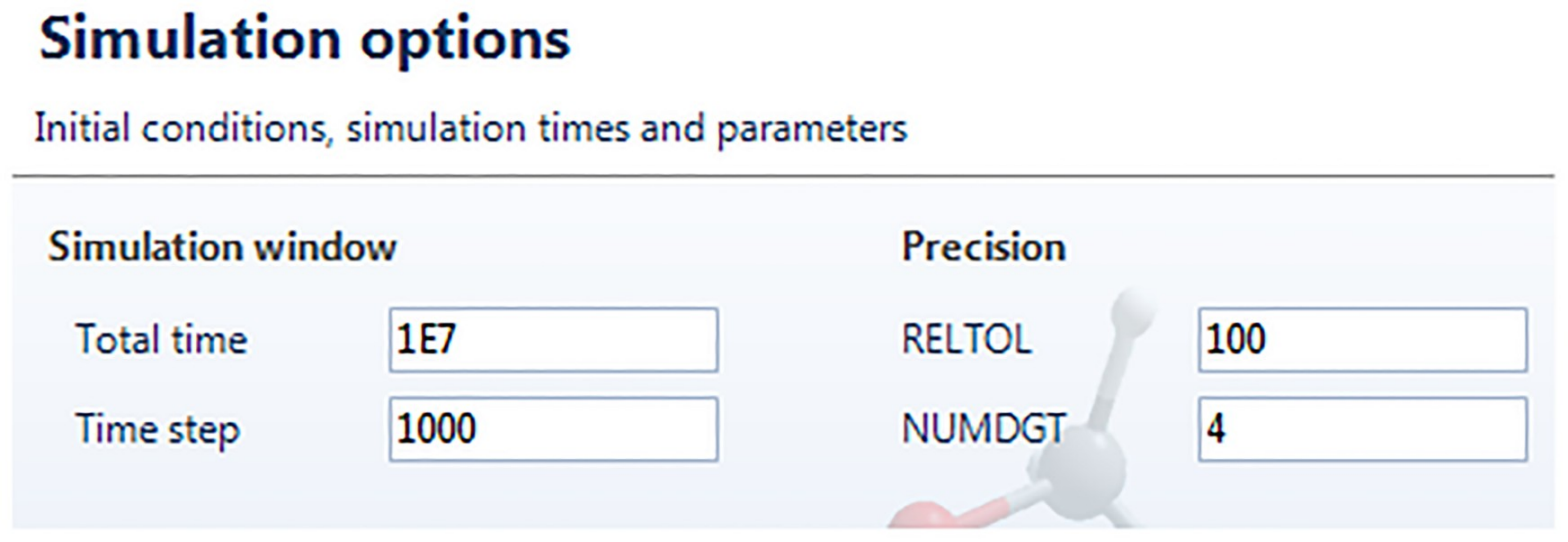
Parameters selection screen for the simulation.

In the next window, “Simulation Options” (see Fig 8), PSpice parameters employed in the simulation are selected. Their meaning can be found by clicking on the help question mark. “Total time” is the time reached by the simulation, in seconds; “Time step” is the print time between data; “RELTOL” adjusts the precision of the numerical algorithm; “NUMDGT” is the number of decimal digits in the output. In the next window, it is shown the “CIR Code” (not shown here), which represents the programming code in PSpice. It is possible to save the CIR by clicking “Save CIR” or load a Pspice out file for the current model by pressing “LoadOUT”.

### Simulation and output data

**Fig 9:**
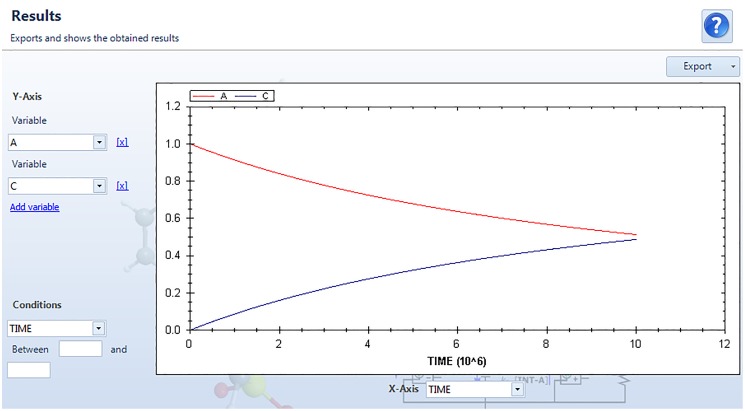
Results window of SimKinet, which displays a graphical environment to plot the data.

The simulation starts by pressing “Next”. Once the model has been successfully solved in PSpice, the window “Results” appears on the screen (Fig 9). This window makes up the graphical environment of SimKinet. In this particular example, the dependence of [**A**] and [**C**] on time until 10^7^ s is shown. Finally, we can click on the “Export” menu (upper right zone) to export data in diverse formats to suit the user. It is possible to export the data in Excel format by clicking on “Generate Excel file” under the label “Filtered data”, and handle the file with advanced software such as Origin.

## Applications

In this section, we will show two practical cases of SimKinet involving researching and academic interest. The first one adresses a problem without analytical solution, guided by the Chapman mechanism for the formation and decomposition of atmospheric ozone. This example may be particularly useful in undergraduate Chemistry courses, where it can be employed to study in depth the order of chemical reactions. Furthermore, in the context of non-analytical solutions, SimKinet is a suitable tool for researchers who need to solve complex kinetic schemes [19,52]. The second practical example, the Olsen attractor, constitutes a very interesting illustration of chaotic dynamics. The study of oscillating chemical reactions becomes essential to understand some key aspects of the behaviour of living organisms. In this way, students can be introduced in nonlinear dynamics, a common feature for some crucial chemical reactions. Non-linear and chaotic dynamics are universal, and have special interest in other related subjects such as Physics or Mathematics at undergraduate courses [53]. From the researching point of view, SimKinet simplifies the determination of chaotic patterns, as for example the insight of chaos in phase diagrams for Josephson junctions [41].

### Non-analytic differential equations associated to a chemical scheme

Even when dealing with simple chemical schemes, we can find a set of differential equations that cannot be solved analytically or be difficult to solve. In these situations, it is customary to carry out theoretical approximations, such as the SSA (steady stationary approximation) or the RSL (rate limiting step) approximation [15]. Even where applicable, they restrict the range of validity for the solutions. Consequently, to obtain accurate results, and without restrictions, numerical approaches become more convenient.

Nowadays, the RKM has been improved to include variable step-size and other numerical features, but it is still found to be an inefficient method when dealing with large numerical simulations, including the dynamics of complex soft matter systems in physics, or complex kinetic chemical schemes [54]. More efficient numerical procedures have been developed along the years, such as the Bulirsch-Stöer method or integration packages in software such as Mathematica or MATLAB [46]. Nevertheless, the user needs to develop programming skills. SimKinet becomes an efficient alternative to these approaches due to its ease of use and power of calculus.

**Fig 10:**
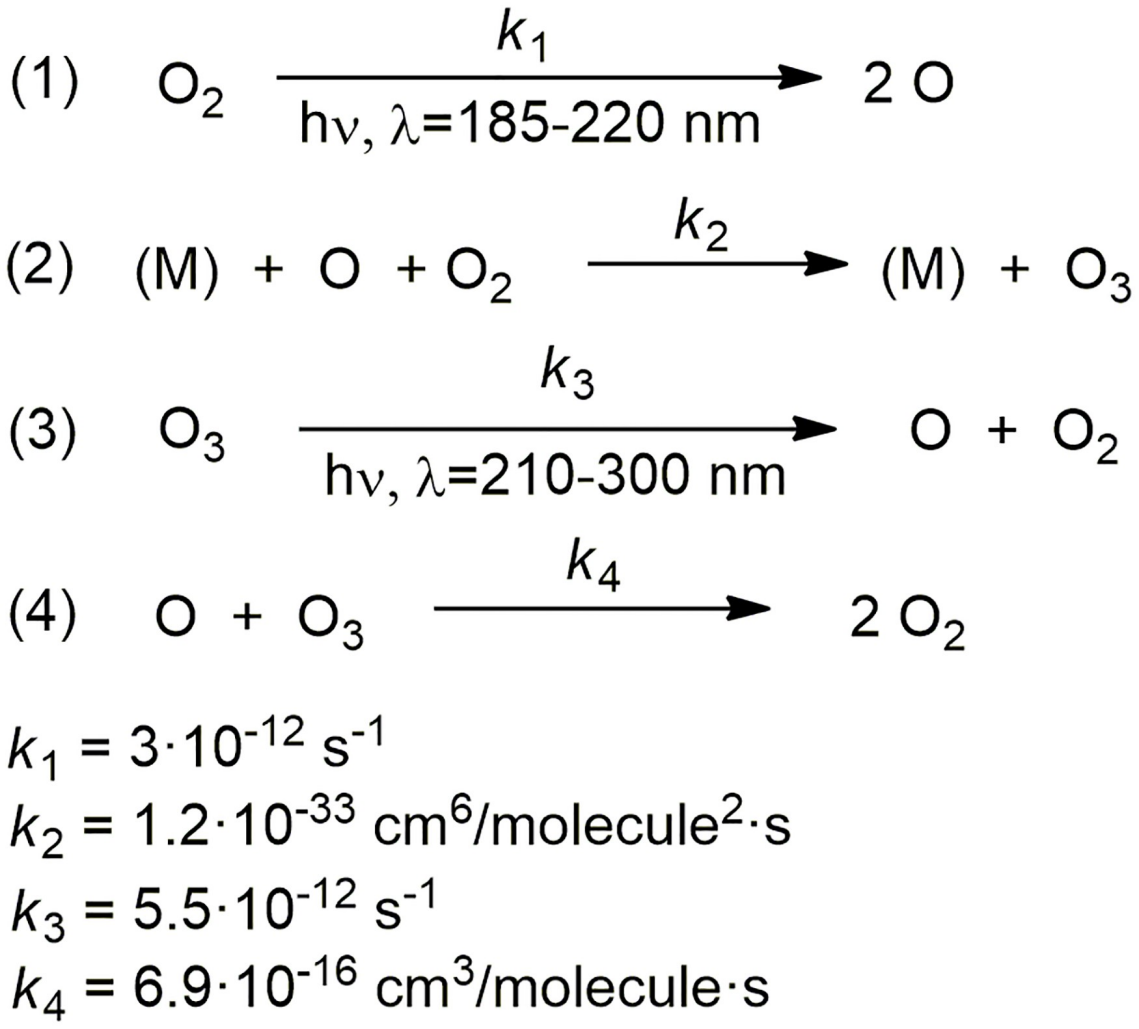
Chapman mechanistic proposal for the photochemical destruction of stratospheric ozone showing the rate constants of each step.

An interesting non-analytic example, in which is common to employ the SSA, is the formation and destruction of stratospheric ozone [55]. A determined concentration of ozone in the stratosphere is vital for life in the Earth as the ozone layer, placed around 25 km of altitude, absorbs the ultraviolet light. To explain the steady state concentration of ozone in the ozone layer, Chapman proposed an approximation to the reaction mechanism involving the cycle of reactions outlined in Fig 10. The principal reaction for ozone production is the recombination of oxygen atoms with O_2_ molecules, steps 1 and 2, whereas steps 3 and 4 account for its destruction. **M** represents a chemical species, usually molecular oxygen or molecular nitrogen, which stabilizes the ozone by absorbing as kinetic energy that which is given off when the step 2 occurs.

The system of differential kinetic equations associated to this mechanism is described by the following set of coupled differential equation, which presents no analytical solution. It is usually solved making use of the SSA for the concentration of intermediate species [9]:

$$\begin{array}{l} \frac{\text{d}\left[{\text{O}}_{2}\right]}{\text{d}t}=-k_{1}\left[{\text{O}}_{2}\right]-k_{2}\left[\text{M}\right]\left[\text{O}\right]\left[{\text{O}}_{2}\right]+k_{3}\left[{\text{O}}_{3}\right]+2k_{4}\left[\text{O}\right]\left[{\text{O}}_{3}\right]\\ \frac{\text{d}\left[\text{O}\right]}{\text{d}t}=2k_{1}\left[{\text{O}}_{2}\right]-k_{2}\left[\text{M}\right]\left[\text{O}\right]\left[{\text{O}}_{2}\right]+k_{3}\left[{\text{O}}_{3}\right]-k_{4}\left[\text{O}\right]\left[{\text{O}}_{3}\right]\\ \frac{\text{d}\left[{\text{O}}_{3}\right]}{\text{d}t}=k_{2}\left[\text{M}\right]\left[\text{O}\right]\left[{\text{O}}_{2}\right]-k_{3}\left[{\text{O}}_{3}\right]-k_{4}\left[\text{O}\right]\left[{\text{O}}_{3}\right] \end{array}$$ (16)

**Fig 11:**
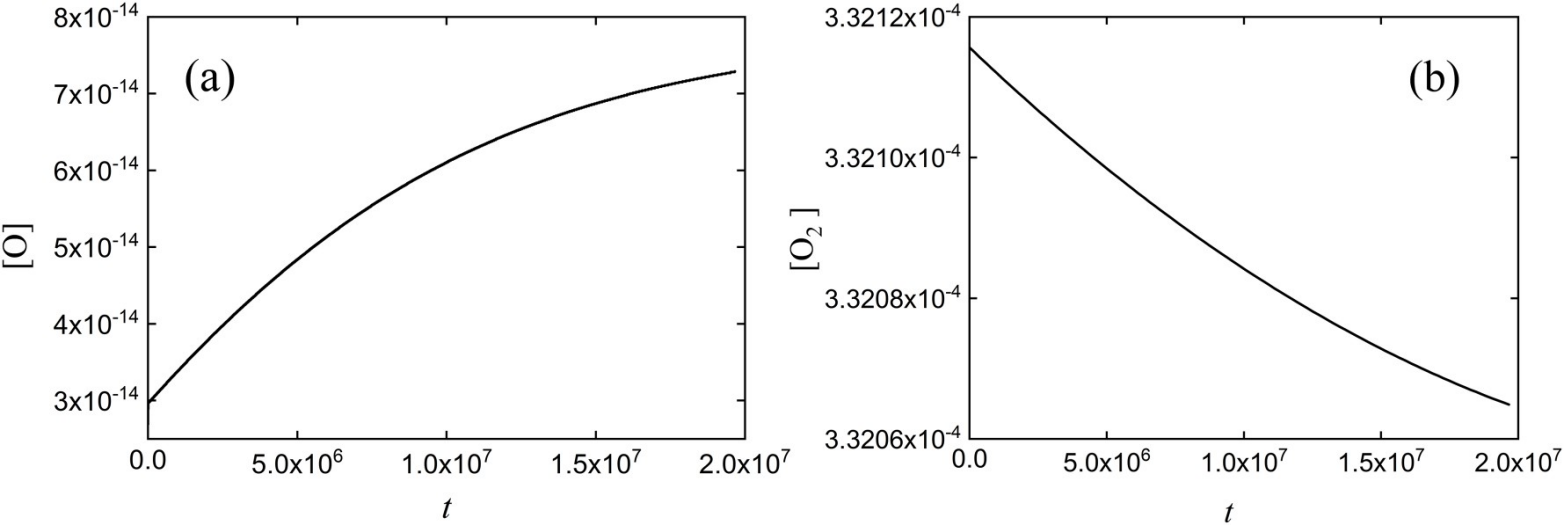
Dependence of (a) [O] and (b) [O_2_] on time corresponding to the simplified Chapman mechanism.

**Fig 12:**
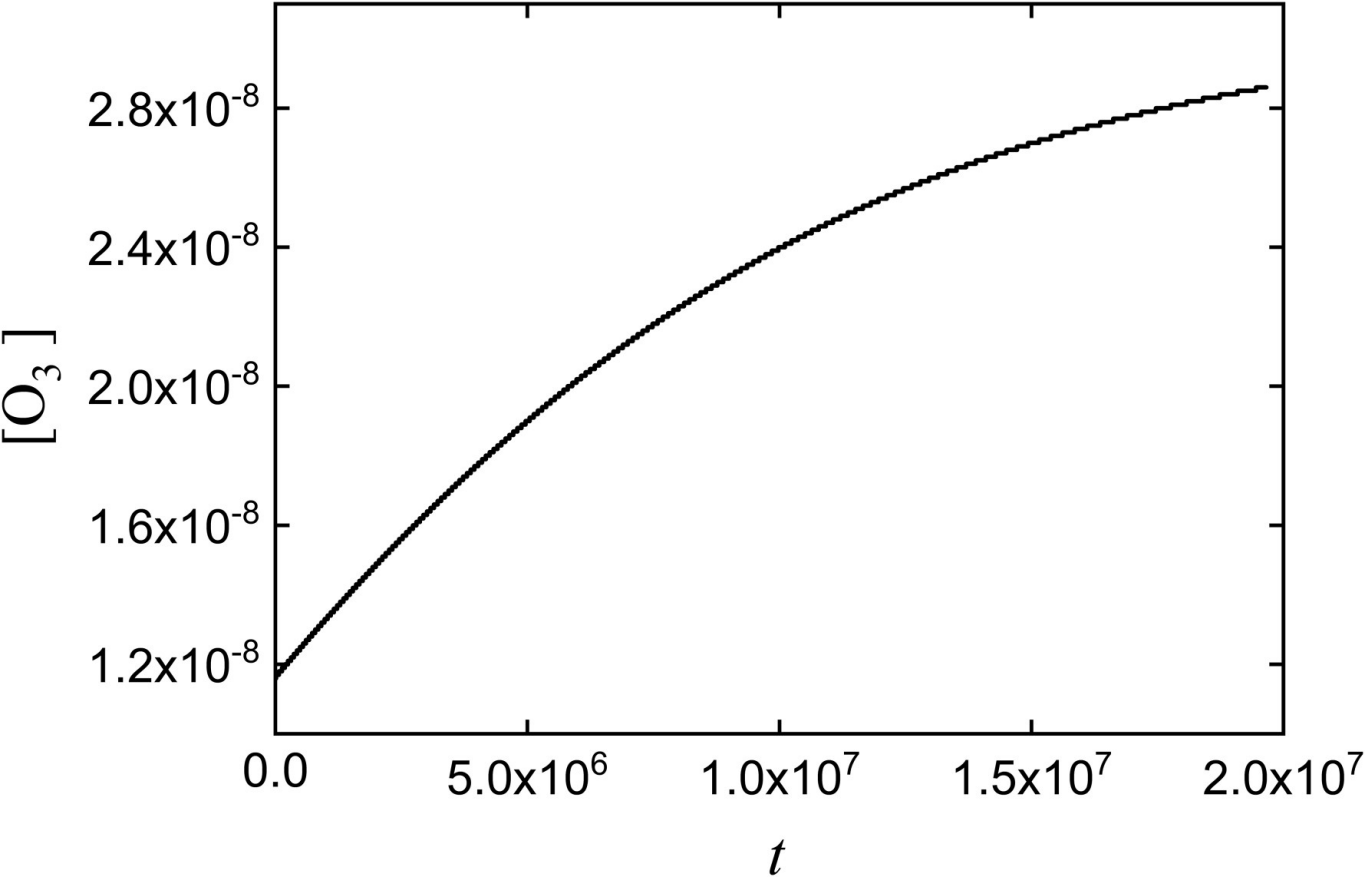
Dependence of [O_3_] on time corresponding to the simplified Chapman mechanism.

**Fig 13:**
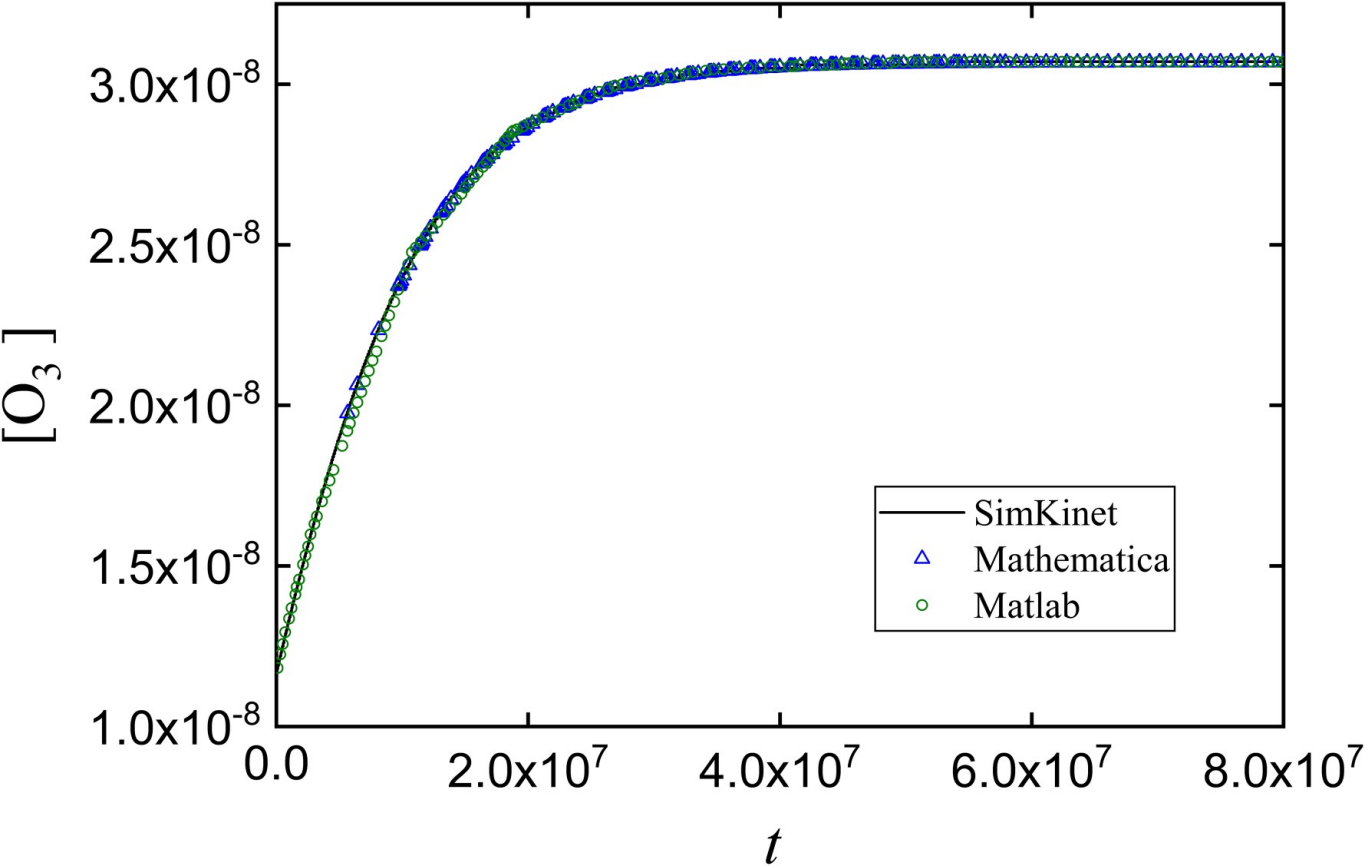
Comparison of the dependence of [O_3_] on time for the stationary state computed via SimKinet (solid line), Mathematica (blue empty triangles) and MATLAB (green empty dots).

Suppose we are interested in determining the change of concentrations of the involved species over time. By using SimKinet, and entering the appropriated data for the initial concentrations of each species [56,57], we solve this set of equations and obtain the following solution for concentrations [O_2_], [O] and [O_3_] up to 10^7^s, shown in Figs 11, 12 and 13:

To test the validity of solutions, we have compared SimKinet results with those obtained via powerful numerical routines implemented in software Mathematica and MATLAB. To this aim, in Fig 13 it is shown the dependence of [O_3_] on time up to 8 × 10^7^ s obtained from SimKinet (solid line), Mathematica (blue empty triangles) and Matlab (green empty dots). It can be inferred by comparing the different plots that the three methods lead to equivalent results.

As can be derived from the above plots, [O] and [O_3_] are monotonically increasing functions within the time range considered, while [O_2_] is monotonically decreasing. The stationary regime for species O_3_ is shown in Fig 13, yielding a stationary value [O_3_] = 3.06 × 10^−8^ mol/l. By contrast, applying the SSA for solving Eq (6), assuming *d*[O]/*dt* = 0, yields [O_3_] = 6.51 × 10^−9^ mol/l, which indicates that the approximation fails for this problem. In general, SimKinet allows the user to test the validity of theoretical approximations in mathematical models described by differential equations.

### Chaotic dynamics: The Olsen attractor

The discovery of oscillating reactions aroused the interest of the scientific community in the last century. Two classic examples are the Lotka-Volterra oscillator and the Belousov-Zhabotinsky reactions [58–60]. Both have been studied extensively, showing oscillating concentrations of reactants. Oscillating chemical reactions constitute an important area of study, becoming essential to understand the behaviour of living organisms. In 1978, H. B. Rössler proposed that chaos could be found in a chemical oscillating system open to its surroundings [61]. Early experimental evidence for chaos was reported for an oscillating enzyme reaction: the peroxidase-oxidase reaction [62]. From then, numerous examples of chaotic behaviour have been found in both abstract and real chemical systems [52,60].

In the peroxidase-oxidase reaction the reduced form of nicotinamide adenine dinucleotide (NADH), is oxidized being molecular oxygen the final electron acceptor:

$${\text{O}}_{2}+\text{NADH}+2\;{\text{H}}^{+}\to 2\;{\text{NAD}}^{+}+2{\;\text{H}}_{2}\text{O}$$

**Fig 14:**
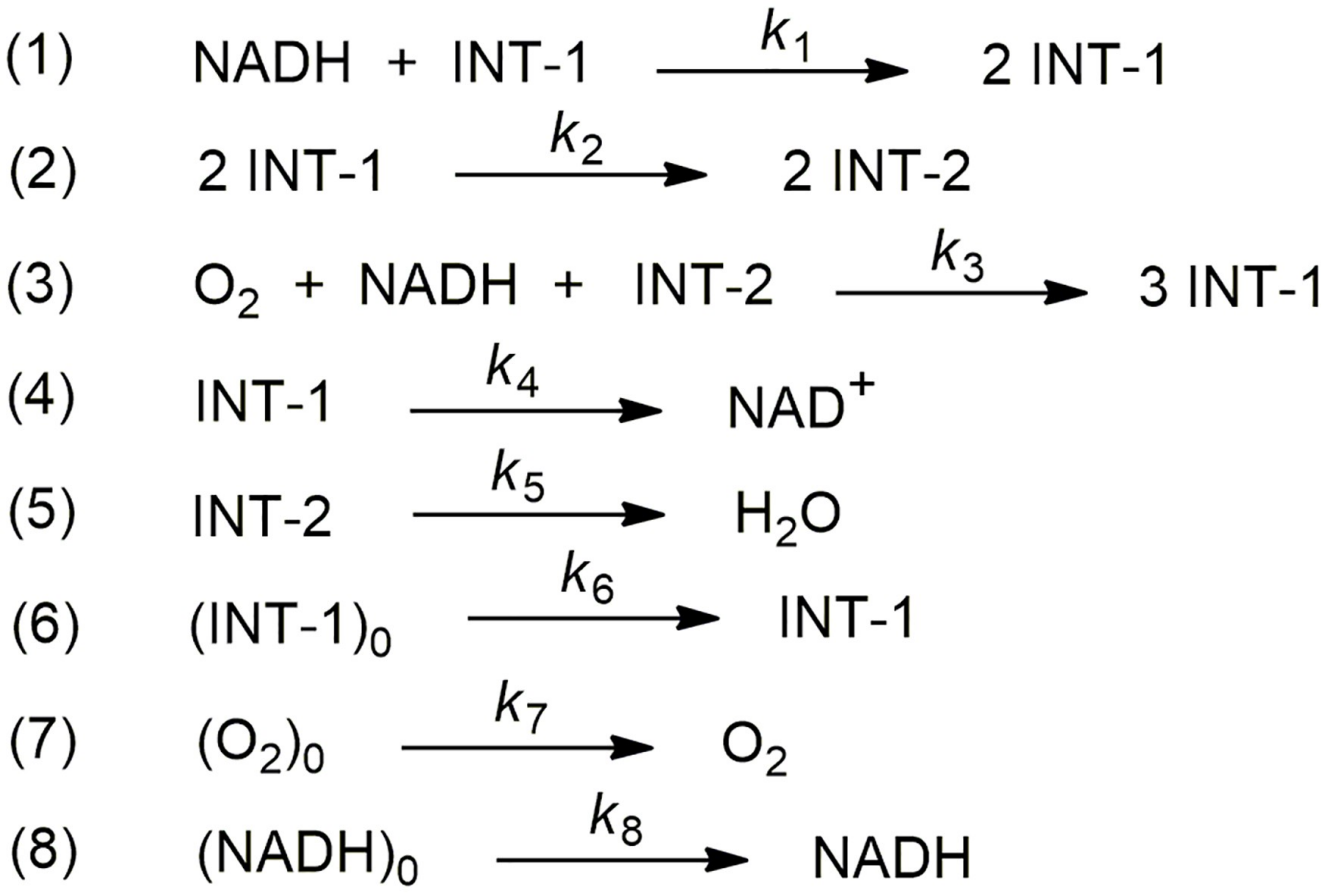
Eight-step simplified mechanism proposed for Olsen for the peroxidase-oxidase reaction.

This reaction is catalysed by peroxidase enzymes. By continuously supplying NADH and O_2_, and depending on the amount of enzyme present in the reaction mixture, the system will show both periodic and non-periodic oscillations. The mechanism of the peroxidase-oxidase reaction is intricate and involves about twenty individual reactions steps, some of which are not known in detail, not even the rate constants. However, its oscillatory behavior, as well as several of the dynamic features of the experimental system, could be successfully modeled by means of the eight-step simplified mechanism known as the Olsen model (see Fig 14).

T he classical Olsen model outlined in Fig 14 describes the nonlinear dynamics of the peroxidase-oxidase reaction, showing both periodic and chaotic attractors [63]. INT-1 and INT-2 are radical intermediates. Note that reaction 6 in Fig 14 involve the spontaneous formation of the free radical intermediate INT-1, which is indispensable for the start of the reaction. Step 7 represents the diffusion of molecular oxygen from the gas phase into the liquid one, whereas step 8 accounts for the infusion of NAHD. Consequently, the associated set of differential equations would be:

$$\begin{array}{l} \frac{\text{d}\left[O_{2}\right]}{\text{d}t}=-k_{3}\left[O_{2}\right]\left[NADH\right]\left[INT-2\right]+k_{7}\left({\left[O_{2}\right]}_{0}-\left[O_{2}\right]\right)\\ \frac{\text{d}\left[NADH\right]}{\text{d}t}=-k_{1}\left[NADH\right]\left[INT-1\right]-k_{3}\left[O_{2}\right]\left[NADH\right]\left[INT-2\right]+k_{8}{\left[NADH\right]}_{0}\\ \frac{\text{d}\left[INT-1\right]}{\text{d}t}=k_{1}\left[NADH\right]\left[INT-1\right]-2k_{2}{\left[INT-1\right]}^{2}+3k_{3}\left[O_{2}\right]\left[NADH\right]\left[INT-2\right]-k_{4}\left[INT-1\right]+k_{6}{\left[INT-1\right]}_{0}\\ \frac{\text{d}\left[INT-2\right]}{\text{d}t}=2k_{2}{\left[INT-1\right]}^{2}-k_{3}\left[O_{2}\right]\left[NADH\right]\left[INT-2\right]-k_{5}\left[INT-2\right] \end{array}$$ (17)

**Fig 15:**
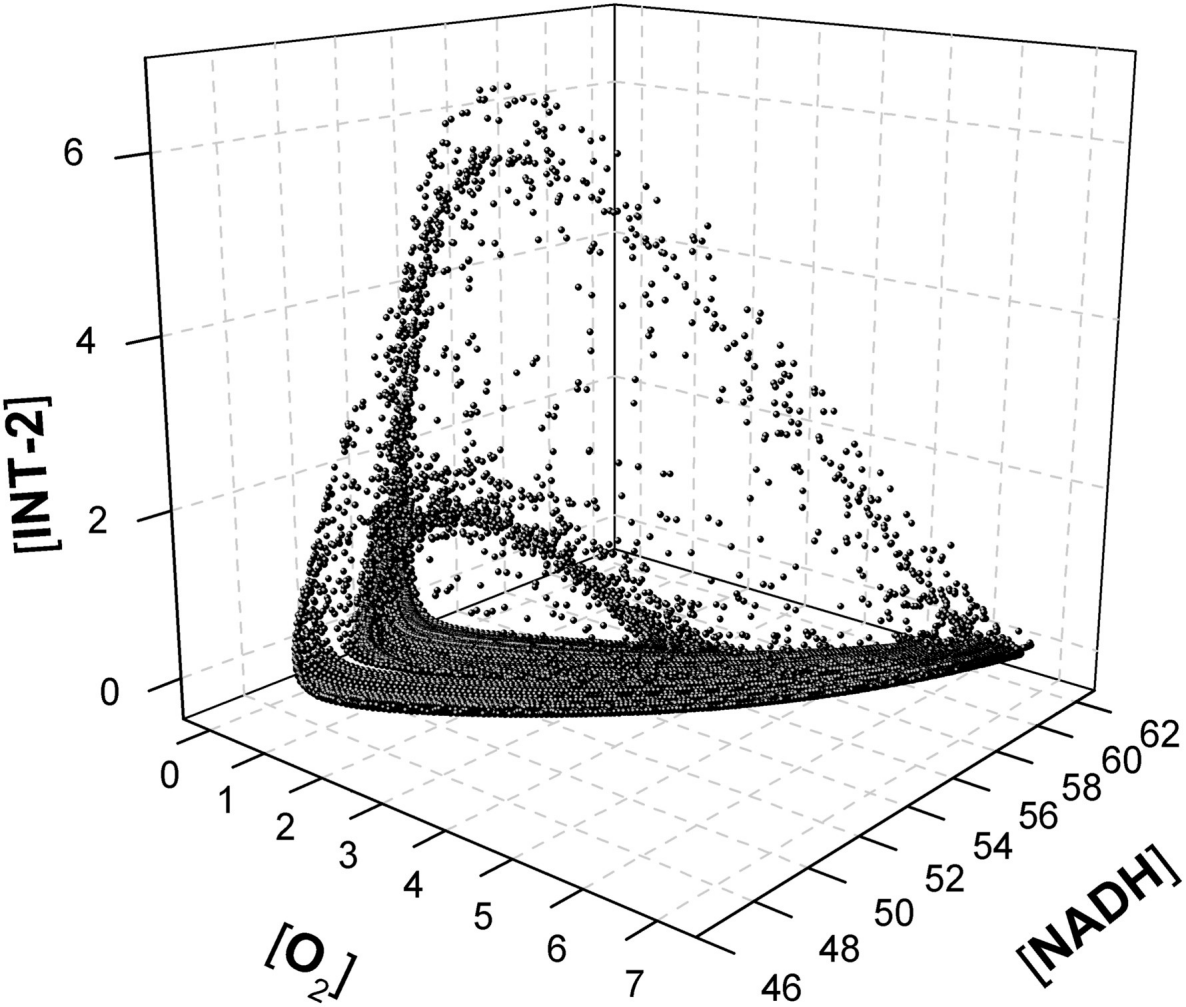
Three dimensional phase space diagram ([O_2_], [NADH] and [INT−1]) of a chaotic attractor for the peroxidase-oxidase reaction. Time and concentrations are dimensionless.

The mathematical model, Eq (17), has been computed in SimKinet (parameters are taken from literature) [63]. The phase space diagram for concentrations [O_2_], [NADH] and [INT−1] are shown in Fig 15, showing a typical chaotic attractor. These results prove that SimKinet becomes a powerful tool to simulate relevant chaotic systems, such as the Rössler attractor, Lotka-Volterra equations, and others [52], and it can help students and researchers to deepen into the mathematical nature of chemical reactions. For example, it is common in some scientific degrees to study oscillating chemical reactions, such as the Belausov-Zhabotinskii or the Briggs-Rauscher [64,65]. The numerical solution of the corresponding mathematical problem via SimKinet allows the user to take control over the variables of the system and to predict the behaviour of the reaction.

## Conclusions

The software SimKinet, a powerful and versatile software, has been designed to solve kinetic chemical equations, and it can be successfully applied for educational and researching purposes. SimKinet is a free and user-friendly tool able to solve a wide range of problems involving differential equations. Its simple handling makes it also suitable for teaching at undergraduate levels without having to resort to theoretical approximations, and allowing the student to deepen into the mathematical nature of scientific models. Two interesting practical examples have been explained to illustrate the capabilities of the software: the Chapman model and the Olsen attractor. Among other interesting uses, it can be employed to determine the range of validity for theoretical approximations, to distinguish between chaotic and periodic behaviour in nonlinear dynamics, or to take control over the product composition in chemical reactions.

## Supporting information

S1 Appendix(Figure A) Connection of flows ${J^{*}}_{R_{i}}$ and J* at the concentration point *R*_*i*_. (Figure B) Electrical network equivalent to Eqs. (S.1)-(S.3).(PDF)

## References

[1] Braun M. Differential Equations and their Applications. 2nd ed New York: Springer-Verlag; 1993.

[2] Margenau H, Murphy GM. The Mathematics of Physics and Chemistry. New Jersey: D. Van Nostrand Company, Inc.; 1968.

[3] Simmons GF. Differential Equations with Applications and Historical Notes. 2nd ed New York: Mc Graw Hill; 1991.

[4] Heicklen J. Atmospheric Chemistry. New York: Elsevier; 2012.

[5] Ullmann’s Fine Chemicals. 1st ed Berlin: Wiley-VCH; 2014.

[6] Jess A, Wasserscheid P. Chemical Technology: An Integral Textbook. Weinheim: Wiley-VCH; 2013.

[7] Weissermel K, Arpe HJ. Industrial Organic Chemistry. Weinheim: Wiley-VCH; 2003.

[8] Bain K, Towns MH. A Review of Research on the Teaching and Learning of Chemical Kinetics Chem Educ Res Pract. 2016; 17: 246–262.

[9] Houston PL. Chemical Kinetics and Reaction Dynamics. New York: Dover Publications; 2012.

[10] Anslyn EV, Dougherty DA. Modern Physical Organic Chemistry. California: University Science Books; 2006.

[11] Isaacs NS. Physical Organic Chemistry. California: Longman Scientific & Technical; 1995.

[12] Wright MR. Introduction to Chemical Kinetics. Chichester: Wiley; 2005.

[13] Metiu H. Physical Chemistry: Kinetics. New York: Garland Science; 2006.

[14] Tyagi P. Chemical Kinetics. New Delhi: Discovery Publishing House Pvt. Ltd; 2006.

[15] Bertrán J, Núñez J. Química Física, vol.2 Barcelona: Ariel; 2002.

[16] Turanyi T, Tomlin AS, Pilling MJ. On the Error of the Quasi-Steady-State Approximation. J Phys Chem. 1993; 97: 163–172.

[17] Tzafriri AR, Edelman ER. On the Validity of the Quasi-Steady State Approximation of Bimolecular Reactions in Solution. J Theor Biol. 2005; 233 (3): 343–350. 10.1016/j.jtbi.2004.10.013 15652144

[18] Viossat V, Ben-Aim J. A Test of the Validity of Steady State and Equilibrium Approximations in Chemical Kinetics. J Chem Educ. 1993; 70: 732–738.

[19] Caravaca M, Sanchez-Andrada P, Soto A, Alajarin M. The Network Simulation Method: a Useful Tool for Locating the Kinetic–Thermodynamic Switching Point in Complex Kinetic Schemes. Phys Chem Chem Phys. 2014; 16: 25409–25420. 10.1039/c4cp02079k 25342168

[20] Horno J. Network Simulation Method. Trivandrum: Research Singpost; 2002.

[21] Lozano-Parada JH, Burnham H, Machuca F. Pedagogical Approach to the Modeling and Simulation of Oscillating Chemical Systems with Modern Software: The Brusselator Model. J Chem Educ. 2018; 95 (5): 758–766.

[22] Kazeroonian A, Fröhlich F, Raue A, Theis FJ, Hasenauer J. CERENA: ChEmical REaction Network Analyzer—A Toolbox for the Simulation and Analysis of Stochastic Chemical Kinetics. PLoS ONE. 2016; 11 (1): e0146732 10.1371/journal.pone.0146732 26807911 PMC4726759

[23] Milanovic JZ, Milanovic P, Kragic R, Kostic M. "Do-It-Yourself" reliable pH-stat device by using open-source software, inexpensive hardware and available laboratory equipment. PLoS ONE. 2018; 13(3): e0193744 10.1371/journal.pone.0193744 29509793 PMC5839570

[24] Ricci RW, Van Doren JM. Using Dynamic Simulation Software in the Physical Chemistry Laboratory. J Chem Educ. 1997; 74 (11): 1372.

[25] Escorihuela F. Determinación del Punto de Switch Cinético Termodinámico en Esquemas Cinéticos Químicos Mediante el Software SimKinet. Final Degree Project. Murcia: University Centre of Defence at the Spanish Air Force Academy; 2015.

[26] García-Sánchez J. Estudio del Problema de Caza-Evasión en Sistemas Autopropulsados Mediante el Software SimKinet. Final Degree Project. Murcia: University Centre of Defence at the Spanish Air Force Academy; 2016.

[27] Chemstations, Inc. CHEMCAD 7 [Internet]. 2018. http://www.chemstations.com/.

[28] Reaction Design. CHEMKIN [Internet]. 2015. http://www.reactiondesign.com/products/chemkin/chemkin-2/.

[29] Slashdot Media. CheMecher [Internet]. 2018. https://sourceforge.net/projects/chemecher/reviews.

[30] Slashdot Media. JKinetics [Internet]. 2018. https://sourceforge.net/projects/jkinetics/.

[31] Bililite.com. Tenua [Internet]. 2018. http://bililite.com/tenua/.

[32] Wolfram. Mathematica [Internet]. 2019. http://www.wolfram.com/mathematica/.

[33] The MathWorks Inc. MATLAB [Internet]. 2019. https://es.mathworks.com/products/matlab.html.

[34] Goldstein H, Poole CP, Safko JL. Classical Mechanics, 3^rd^ ed San Francisco: Addison Wesley; 2002.

[35] Bishop RH. Mechatronics: An Introduction. Florida: CRC Press; 2005.

[36] Marion JB. Classical Dynamics of Particles and Systems. London: Academic Press; 2013.

[37] Tipler PA, Mosca G. Physics for Scientists and Engineers: Extended Version. New York: W. H. Freeman; 2003.

[38] Olson HF. Dynamical Analogies. New York: Van Nostrand; 1943.

[39] Moya AA. A Ladder Network Modelling the Electrochemical Impedance of the Diffusion and Reaction Processes in Semi-Infinite Space. Phys Chem Chem Phys. 2016; 18: 3812–3816. 10.1039/c5cp07476b 26763107

[40] Serna J, Velasco F, Soto-Meca A. Application of Network Simulation Method to Viscous Flows: The Nanofluid Heated Lid Cavity Under Pulsating Flow. Comput Fluids. 2014; 91: 10–20.

[41] Blackburn JA, Smith HJT, Gronbech-Jensen N. Resonant Steps in the Characteristics of a Josephson Junction Coupled to a Transmission Line. J Appl Phys. 1991; 70: 2395–2401.

[42] Kozuch S, Martin JML. The Rate-Determining Step is Dead. Long Live the Rate-Determining State! Chem Phys Chem. 2011; 12: 1413–1418. 10.1002/cphc.201100137 21523880

[43] Laidler K J. Rate-Controlling Step: A Necessary or Useful Concept? J Chem Educ 1988; 65: 250–254.

[44] Murdoch JR. What is the Rate-Limiting Step of a Multistep Reaction? J Chem Educ 1981; 58: 32–36.

[45] Campbell CT. The Degree of Rate Control: A Powerful Tool for Catalysis Research. ACS catal. 2017; 7: 2770–2779.

[46] Press WH, Teukolsky SA, Vetterling WT, Flannery BP. Numerical Recipes 3rd Edition: The Art of Scientific Computing New York: Cambridge University Press; 2007.

[47] OrCAD. OrCAD PSpice Designer [Internet]. 2018. http://www.orcad.com/.

[48] Moya AA. Theory of the Formation of the Electric Double Layer at the Ion Exchange Membrane–Solution Interface. Phys Chem Chem Phys. 2015; 17: 5207–5218. 10.1039/c4cp05702c 25600122

[49] Gimeno F, Caravaca M, Soto A, Vera JA, Guirao JL, Fernández-Martínez M. Applying the Network Simulation Method for Testing Chaos in a Resistively and Capacitively Shunted Josephson Junction Model. Results Phys. 2017; 7: 813–822.

[50] Truhlar DG, Garrett BC, Klippenstein SJ. Current Status of Transition-State Theory. J Phys Chem. 1996; 100: 12771–12800.

[51] Cabaleiro-Lago EM, Rodríguez-Otero J, González-López I, Peña Gallego A, Hermida-Ramón JM. A DFT Study of the Pericyclic/Pseudopericyclic Character of Cycloaddition Reactions of Ethylene and Formaldehyde to Buta-1,3-dien-1-one and Derivatives. J Phys Chem A. 2005; 109: 5636–5644. 10.1021/jp050624x 16833896

[52] Strogatz SH. Nonlinear Dynamics and Chaos: With Applications to Physics, Biology, Chemistry, and Engineering. 2nd ed Boca Raton: Taylor & Francis Group; 2018.

[53] Yang XS, Li Q. A Computer-Assisted Proof of Chaos in Josephson Junction. Chaos Soliton Frac. 2006; 27: 25–30.

[54] Gustafsson K. Control-Theoretic Techniques for Stepsize Selection in Implicit Runge-Kutta Methods. ACM Trans Math Softw. 1994; 20 (4): 496–517.

[55] Johnston H. S. Atmospheric Ozone. Ann Rev Phys Chem. 1992; 43: 1–32. 18338974 10.1146/annurev.pc.43.100192.000245

[56] Warneck P. Chemistry of the Natural Atmosphere. London: Elsevier Science; 1988.

[57] Sander SP, Friedl RR, DeMore WB, Ravishankara A, Kolb CE. Chemical Kinetics and Photochemical Data for Use in Stratospheric Modeling Supplement to Evaluation 12: Update of Key Reactions, vol. 97–4 California: JPL publication; 1997.

[58] Lotka A. Contribution to the Theory of Periodic Reactions. J Phys Chem. 1910; 14 (3): 271–274.

[59] Goel NS, Maitra SC, Montroll EW. On the Volterra and Other Nonlinear Models of Interacting Populations Nonlinear Models in Interacting Populations. New York: Academic Press; 1971.

[60] Epstein IR, Showalter K. Nonlinear Chemical Dynamics: Oscillations, Patterns, and Chaos. J Phys Chem. 1996; 100 (31): 13132–13147.

[61] Rössler OE. Chaotic Behavior in Simple Reaction Systems. Z Naturforsch. 1976; 31: 259–264.

[62] Olsen LF, Degn H Chaos in an Enzyme Reaction. Nature. 1977; 267: 177–178. 16073439 10.1038/267177a0

[63] Olsen LF. An Enzyme Reaction with a Strange Attractor. Phys Lett A. 1983; 94: 454–457.

[64] Strizhak P, Menzinger M. Stirring Effect on the Bistability of the Belousov–Zhabotinsky Reaction in a CSTR. J Phys Chem. 1996; 100 (49): 19182–19186.

[65] Briggs TS, Rauscher WC. An Oscillating Iodine Clock. J Chem Ed. 1973; 50 (7): 496.

