# Supplementary material for: SimKinet: A free educational tool based on an electrical analogy to solve chemical kinetic equations
Source: PLoS One. 2019 Mar 8;14(3):e0213302. doi: 10.1371/journal.pone.0213302 (PMC6407758; doi:10.1371/journal.pone.0213302)
Supplement: S1 Appendix — (Figure A) Connection of flows J*Ri and J* at the concentration point Ri. (Figure B) Electrical network equivalent to Eqs. (S.1)-(S.3). (PDF) [file pone.0213302.s001.pdf]

S1 Appendix

NSM electrical analogy applied to a prototypical chemical reaction

M. Caravaca, P. Sanchez-Andrada,
and A. Soto-Meca

Let us consider the example of the prototypical reaction

$$\mathbf{A} \underset{k_2}{\overset{k_1}{\rightleftarrows}} \mathbf{B} + \mathbf{C}$$

Provided that k_1 and k_2 are, respectively, the forward and back rate constants for the reaction, the set of differential equations associated to the chemical scheme is:

$$\frac{\mathrm{d}[\mathbf{A}]}{\mathrm{d}t} + (k_1 + k_2)[\mathbf{A}] - k_{-1}[\mathbf{B}] - k_{-2}[\mathbf{C}] = 0. \tag{S.1}$$

$$\frac{\mathrm{d}[\mathbf{B}]}{\mathrm{d}t} - k_1[\mathbf{A}] + k_{-1}[\mathbf{B}] = 0. \tag{S.2}$$

$$\frac{\mathrm{d}[\mathbf{C}]}{\mathrm{d}t} - k_2[\mathbf{A}] + k_{-2}[\mathbf{C}] = 0. \tag{S.3}$$

We focus on the first equation, Eq. (S.1), corresponding to reactant **A**. The first derivative is modeled through a condenser of 1 F. Concentration [**A**] is always a positive quantity so, if we choose a ground node at one end of the condenser to design the equivalent circuit, the branch current J^*_{A} is always outgoing from a particular node (see Figure A). This node is labeled as **A** and its voltage is just the voltage of the condenser (which is equivalent to the concentration of the reactant, [**A**]). Since the first derivative has units of current, the rest of the addends in the equation also have it, being all of them represented by the flow J^*. Each addend has to be placed in the electric circuit as a parallel branch to satisfy KCL. Due to the fact that all the species concentrations are positive, also all the node voltages are, and the mathematical sign of each addend determines if the branch current is incoming or outgoing. In the particular case of Eq. (S.1), the second addend is equivalent to an outgoing current, while the third and fourth addends correspond to incoming currents, as show in Figure B.

In general, flows J^* and $J^*_{R_i}$ satisfy KCL at a point of the circuit called node i, or equivalent, R_i (see Figure A).

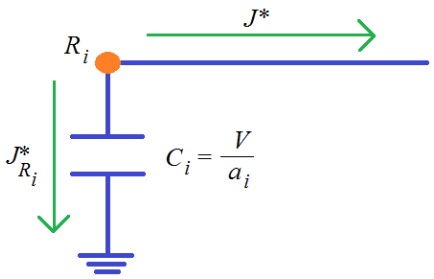

Figure A. Connection of flows $J^*_{R_i}$ and J^* at the concentration point R_i.

It remains to model, for each differential equation, the addends which represent the flow term J^* (different from the first derivatives), employing appropriate electric devices. In general, these addends are functions of the node voltages, even including the proper node voltage of the circuit, so they can present coupled terms between equations. The electric device known as voltage-controlled current source is a standard device in circuit analysis, defined as a current source whose value depends on a function of other voltages somewhere else in the network. The addends which conform the flow term J^* are, in general, functions of the node voltages of the whole network, so they can be easily implemented by the voltage-controlled current sources computed in a software such as Pspice. It is common to model each addend of a particular differential equation as a parallel current branch containing one of these controlled currents, but it is also possible to summarize all addends in only one voltage-controlled current source. To complete the electrical network, in order to establish the connection between isolated circuits, we define a common ground node (see Figure B), easily implemented in Pspice.

A resistor placed in parallel inside each individual circuit is necessary in order to satisfy criteria of continuity, and it is chosen as very large. It does not model any term of the differential equations.

The generalization of the analogy established by the NSM to multi-step chemical reactions is immediate. The key is to employ again voltage-controlled current sources. In these kind of problems we will have one circuit per species, which contains a capacitor (associated to the time derivative) and as many controlled current sources as addends, no matter what kind of dependence have on the voltage nodes of the network. The complete electrical network corresponding to Eqs. (S.1)-(S.3) is shown in Figure B.

References

[1] J. Horno, *Network simulation method*, Research Singpost, Trivandrum, 2002.

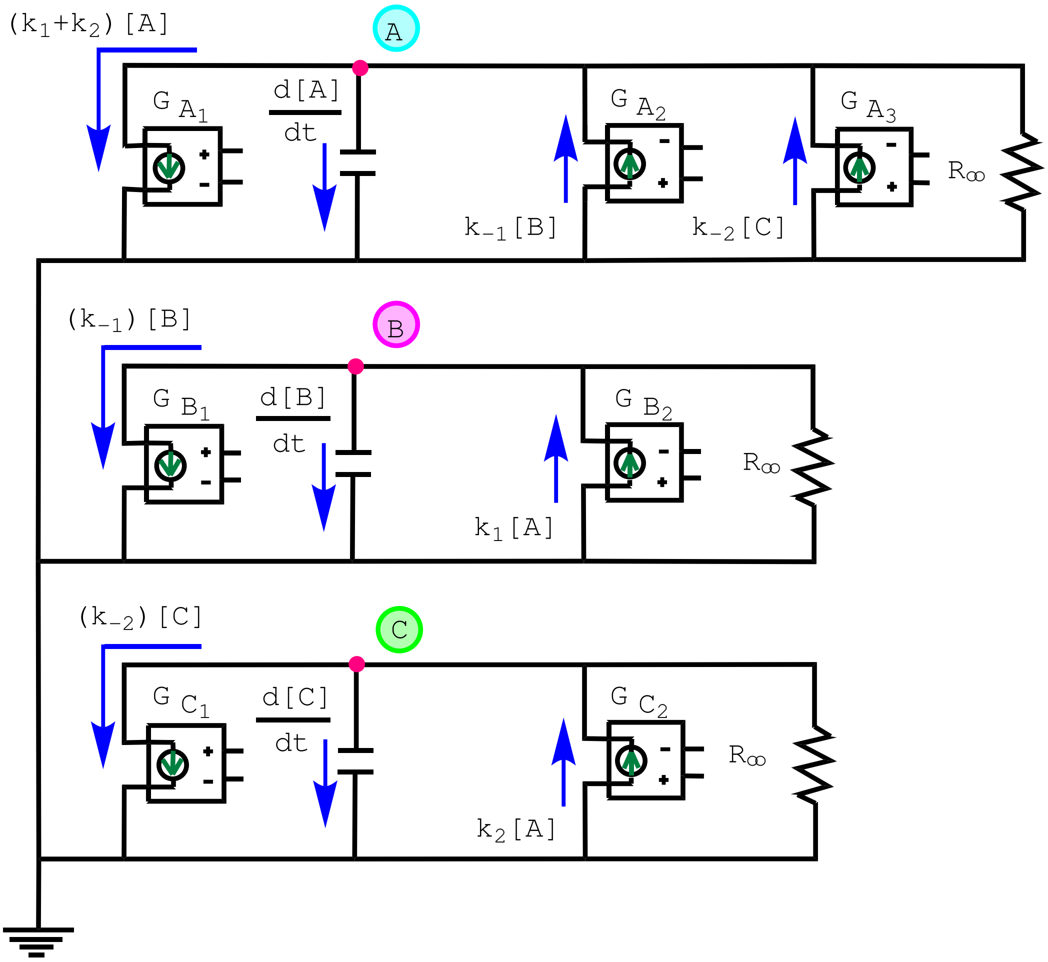

Figure B. Electrical network equivalent to Eqs. (S.1)-(S.3).
